# Crystal structures of two thia­zolidinone derivatives bearing a tri­chloro­methyl substituent at the 2-position

**DOI:** 10.1107/S2056989018013257

**Published:** 2018-09-28

**Authors:** Ahmed Nuriye, Hemant Yennawar, Kevin Cannon, John Tierney

**Affiliations:** a Department of Chemistry, The Pennsylvania State University, Abington College, 1600 Woodland Road, Abington, Pennsylvania, 19001, USA; b Department of Biochemistry and Molecular Biology, The Pennsylvania State University, University Park, Pennsylvania, 16802, USA; c Department of Chemistry, The Pennsylvania State University, Brandywine Campus, 25 Yearsley Mill Road, Media, Pennsylvania, 19063, USA

**Keywords:** crystal structure, 1,3-thia­zolidin-4-ones, tri­chloro­meth­yl

## Abstract

The title compounds 2-tri­chloro­methyl-3-phenyl-1,3-thia­zolidin-4-one (C_10_H_8_Cl_3_NOS), **1** and 3-(4-chloro­phen­yl)-2-tri­chloro­methyl-1,3-thia­zolidin-4-one (C_10_H_7_Cl_4_NOS) **2**, are structurally related with one atom substitution difference in the *para* position of the benzene ring. In both structures, the thia­zolidinone ring adopts an envelope conformation with the S atom as the flap. The dihedral angles between the rings [48.72 (11) in **1** and 48.42 (9)° in **2**] are very similar and the mol­ecules are almost superimposable. In both crystal structures, C—H⋯O ‘head-to-tail’ inter­actions between the chiral carbon atoms and the thia­zolidinone oxygen atoms result in infinite monochiral chains along the direction of the shortest unit-cell parameter, namely *a* in **1** and *b* in **2**. C—H⋯π inter­actions between the thia­zolidinone carbon atom at the 4-position and the phenyl ring of the neighboring enanti­omer also help to stabilize the packing in each case, although the crystals are not isostructural.

## Abstract 2

The mol­ecular conformations of the racemic title mol­ecules are almost identical. Each crystal structure features a short C—H⋯O hydrogen bond arising from the chiral carbon atom, which generates monochiral chains, although the overall structures are centrosymmetric.

## Chemical context   

The title compounds **1** and **2** are unique structures containing a tri­chloro­methyl substituent at the 2-position of the thia­zolidinone ring. Their synthesis was first reported as two of only three known 2-alkyl thia­zolidin-4-one compounds (Tierney, 1989 ▸; Issac *et al.*, 1996 ▸). Substituted thia­zolidin-4-one compounds are synthesized by reacting an *in situ* generated imine (Schiff base) with thio­glycolic acid and with a mechanism to remove the water byproduct (Surrey, 1947 ▸; Erlenmeyer & Oberlin, 1947 ▸). Therefore, when chloral is reacted with aryl­amines, the corresponding imine is formed, which, upon reacting with thio­glycolic acid, produces the desired 2-tri­chloro­methyl-3-aryl-thia­zolidin-4-one (Issac *et al.*, 1996 ▸). It is inter­esting to note, however, that the reaction of chloral with some alkyl amines results in an *N*-alkyl­formamide product when the initially formed aminol loses chloro­form instead of water (Mascavage *et al.*, 2010 ▸). The loss of chloro­form appears to be more facile in electron-rich *N*-alkyl­amines that can stabilize the transition state and lower the energy of activation of the elimination step better than the less electron-rich *N*-aryl­amines. On the other hand, imine formation is favored with aryl­amines because of the lower p*K_a_* of the proton on the nitro­gen in the aminol, which facilitates the removal of water to give an imine. As part of our ongoing studies in this area, we now describe the crystal structures of **1** and **2**.
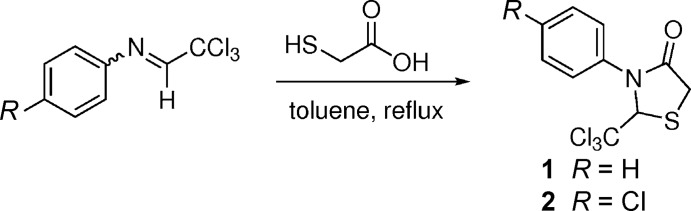


## Structural commentary   

**Figure 1:**
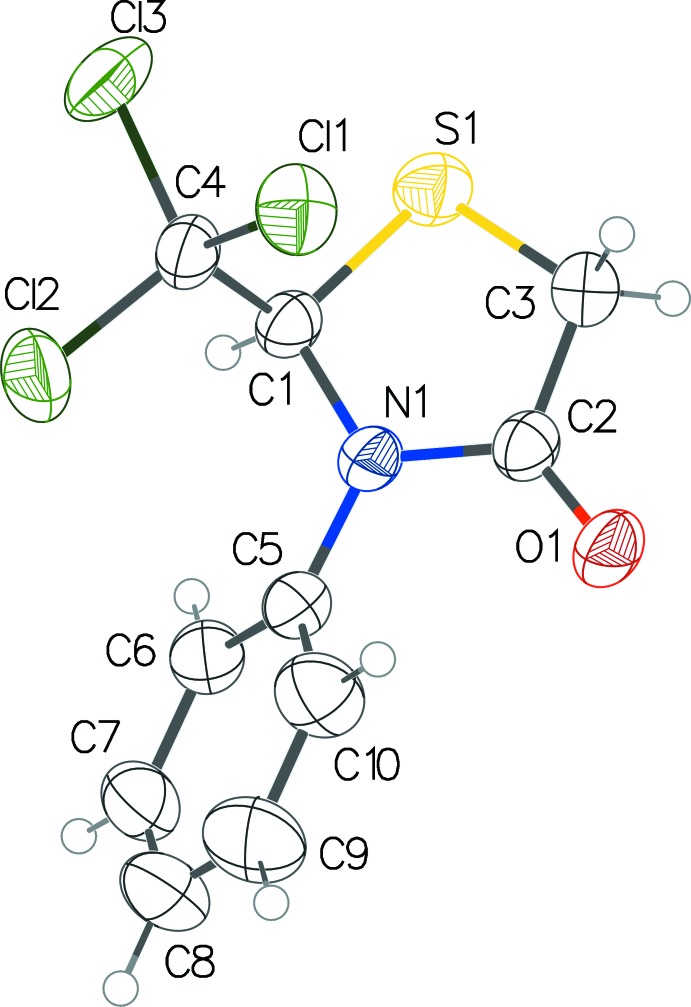
The mol­ecular structure of compound **1** with displacement ellipsoids drawn at the 50% probability level.

**Figure 3:**
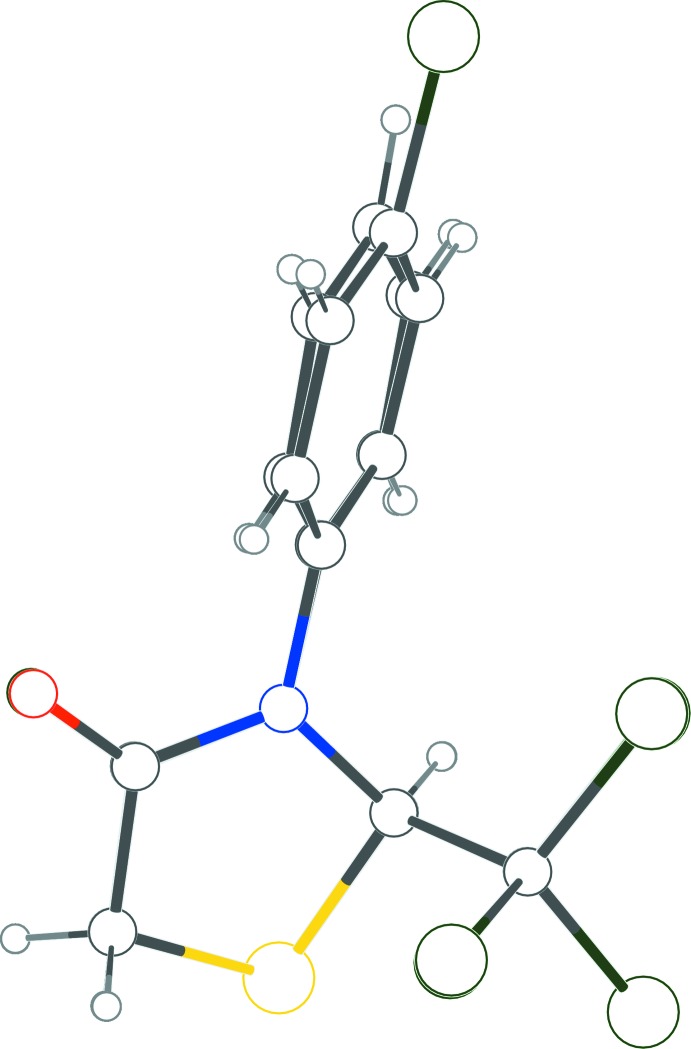
Superposition image for structures **1** and **2** showing similarity of conformation.

Compounds **1** and **2** are structurally related with one atom substitution difference in the *para* position of the benzene ring; a hydrogen atom is substituted for a chlorine atom (Figs. 1 ▸ and 2 ▸). Both contain a stereogenic centre at C1, which is arbitrarily assigned as having an *R* configuration in the asymmetric units of the centrosymmetric unit cells. In both structures, the thia­zolidinone ring adopts an envelope conformation with the S atom as the flap. The sulfur atom is displaced from the thia­zolidinone ring plane by 0.35 (2) Å in both structures. The dihedral angles between the thia­zolidinone and phenyl rings are 48.72 (11) in **1** and 48.42 (9)° in **2**. The C1—N1 and C1—S1 bond lengths are 1.445 (2) Å and 1.816 (2) Å, respectively, for structure **1** and 1.4471 (18) Å and 1.8181 (16) Å, respectively, for structure **2**. The N—C—S bond angle is found to be 106.52 (12)° in structure **1** and 106.23 (10)° in structure **2**. Overall, the molecular structures of both are almost exactly superimposable (Fig. 3 ▸). Bond length and angle values in the thia­zolidinone ring in both structures appear to be typical and match currently available data (Yennawar *et al.*, 2015 ▸).

## Supra­molecular features   

**Figure 4:**
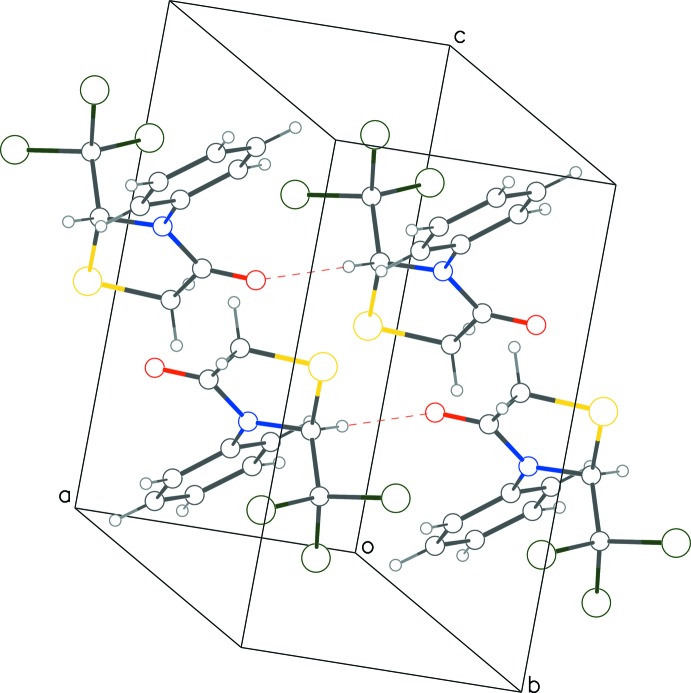
Crystal packing diagram for **1** with red dotted lines for C—H⋯O contacts.

Both extended structures exhibit C—H⋯O ‘head-to-tail’ inter­molecular inter­actions between the chiral carbon atom C1 and the thia­zolidinone oxygen atom (Tables 1 ▸ and 2 ▸; Figs. 4 ▸ and 5 ▸) that result in infinite monochiral chains propagating along the shortest unit-cell dimension, namely *a* in **1** and *b* in **2**; in both cases adjacent mol­ecules are related only by translational symmetry. The short H⋯O distances of 2.30 Å suggest that these inter­actions are relatively strong. Weak C—H⋯π inter­actions between the tetra­hedral, non-chiral carbon atom (C3) of the thia­zolidinone ring and the phenyl ring of the symmetry-related enanti­omer are also observed in both structures (Tables 1 ▸ and 2 ▸). Despite the similar mol­ecular conformations and inter­molecular inter­actions, the crystals are not isostructural (**1** is triclinic and **2** is monoclinic).

## Database survey   

To date, there have been no reported X-ray structures of substituted 2-tri­chloro­methyl-3-phenyl-1,3-thia­zolidin-4-ones or the unsubstituted parent compound. However, there are a number of studies for structures containing aromatic moieties at the 2- and 3-positions of the thia­zolidin-4-one ring (Kumar *et al.*, 2016 ▸; Yennawar *et al.*, 2014 ▸). In addition, there is a structural and conformational study of 3-cyclo­hexyl-2-phenyl-1,3-thia­zolidin-4-one (Cannon *et al.*, 2013 ▸).

## Synthesis and crystallization   

The two compounds were synthesized using previously reported procedures (Tierney, 1989 ▸; Issac *et al.*, 1996 ▸).

2-Tri­chloro­methyl-3-phenyl-1,3-thia­zolidin-4-one (**1**): Yield 43%; m.p. 447–448 K; IR: 1687 cm^−1^; ^1^H NMR: δ 7.1–7.5 (*m*, 5H, aromatics), 5.72 (*s*, *J* = 1.6 Hz, 1H), 3.77–3.96 (*dd*, *J* = 1.6, 14.1 Hz, 2H); ^13^C NMR: δ 171.65 (C=O), 138.45 (N—Ar), 129.17, 127.98, 126.98, 103.18 (CC13), 77.69 (C2), 33.08 (C5). Analysis calculated for C_10_H_8_NOSC1_3_: C, 40.40; H, 2.72; N, 4.72; Cl, 35.86. Found: C, 40.60, H, 2.74; N, 4.60; Cl, 35.44.

2-Tri­chloro­methyl-3-(4-chloro­phen­yl)-1,3-thia­zolidin-4-one (**2**): Yield 20%; mp 456–458 K; IR: 1685 cm^−1^; ^1^H NMR: δ 7.11–7.50 (*m*, 4H, aromatics), 6.04 (*s*, *J* = 1.2 Hz, 1H), 3.80–3.92 (*dd*, *J* = 1.2, 15.9 Hz, 2H); ^13^C NMR: δ 171.61 (C=O), 136.96 (N—Ar), 133.78 (C—CI), 129.46, 127.92, 103.06 (CCI3), 77.51 (C2), 32.65 (C5). Analysis calculated for C_10_H_7_NOSC1_4_: C, 36.47; H, 2.13; N, 4.25. Found: C, 36.65; H, 2.12; N, 4.04.

Compound **1** was crystallized by vapor diffusion where the sample was dissolved in acetone and placed in a chamber containing hexa­nes. Compound **2** was crystallized by the same method using methyl­ene chloride as the solvent and a chamber containing hexa­nes.

## Refinement   

Crystal data, data collection and structure refinement details are summarized in Table 3 ▸. The hydrogen atoms were placed in calculated positions with C—H = 0.93–0.98 Å and refined using a riding model with fixed isotropic displacement parameters: *U*_iso_(H) = 1.5*U*_eq_(C) for the methyl group and *U*_iso_(H) = 1.2*U*_eq_(C) for the remaining H atoms.

## Supplementary Material

Crystal structure: contains datablock(s) I, 2, 1. DOI: 10.1107/S2056989018013257/hb7769sup1.cif

Supporting information file. DOI: 10.1107/S2056989018013257/hb77691sup2.cml

Supporting information file. DOI: 10.1107/S2056989018013257/hb77692sup3.cml

CCDC references: 1868299, 1868298

Additional supporting information:  crystallographic information; 3D view; checkCIF report

## Figures and Tables

**Figure 2:**
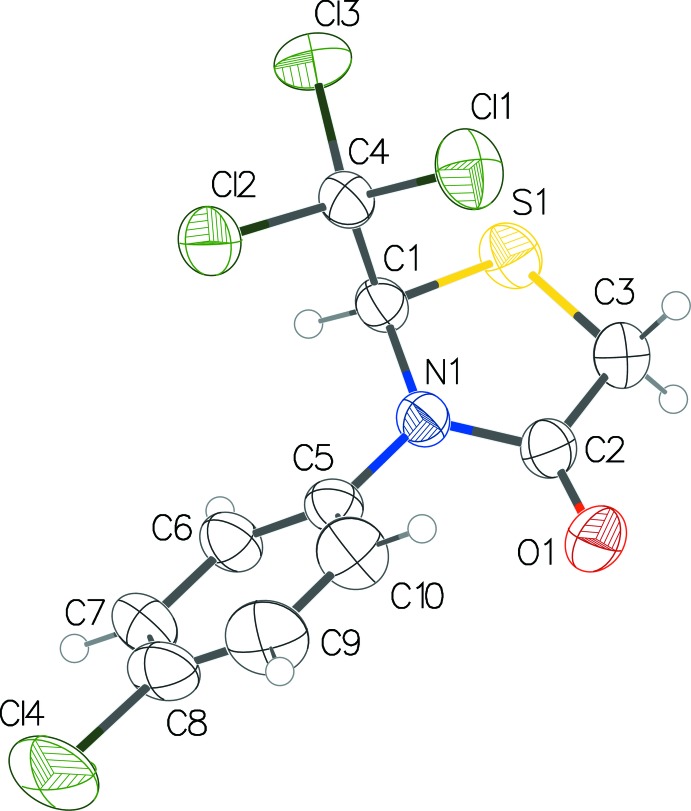
The mol­ecular structure of compound **2** with displacement ellipsoids drawn at the 50% probability level.

**Figure 5:**
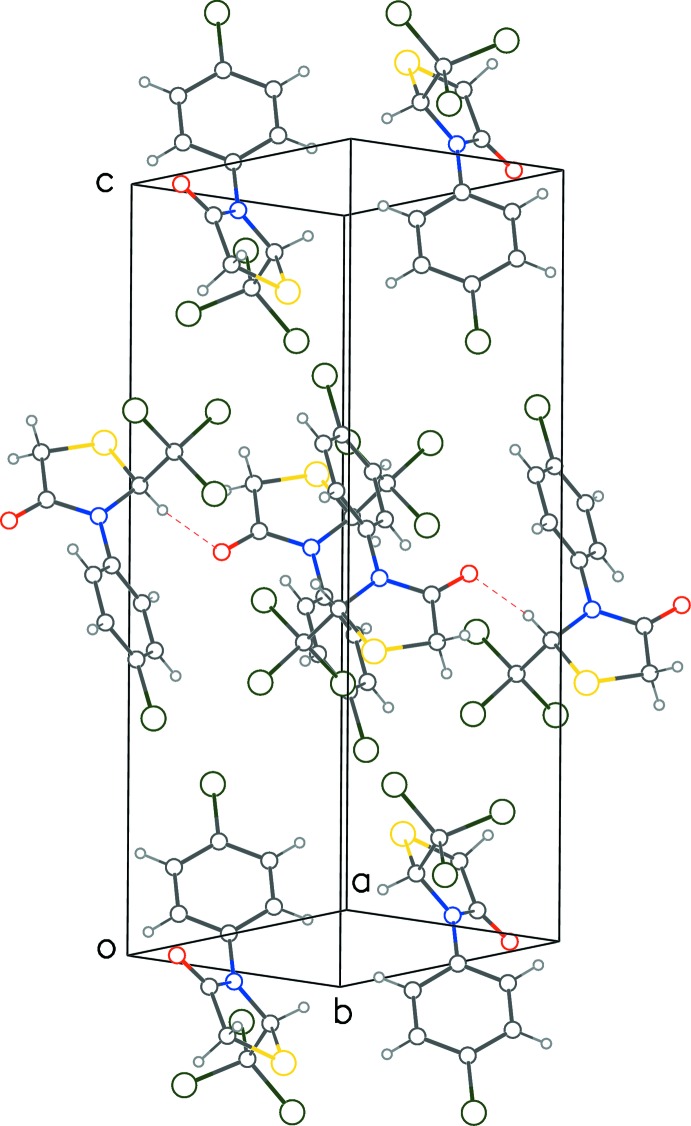
Crystal packing diagram for **2** with red dotted lines for C—H⋯O contacts.

**Table 1:** Hydrogen-bond geometry (Å, °) for **1** [Chem scheme1] *Cg*2 is the centroid of the C5–C10 ring

*D*—H⋯*A*	*D*—H	H⋯*A*	*D*⋯*A*	*D*—H⋯*A*
C1—H1⋯O1^i^	0.98	2.30	3.251 (2)	164
C3—H3*A*⋯*Cg*2^ii^	0.97	2.79	3.748 (2)	170

Symmetry codes: (i) 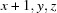; (ii) 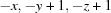.

**Table 2:** Hydrogen-bond geometry (Å, °) for **2** [Chem scheme1] *Cg*2 is the centroid of the C5–C10 ring

*D*—H⋯*A*	*D*—H	H⋯*A*	*D*⋯*A*	*D*—H⋯*A*
C1—H1⋯O1^i^	0.98	2.30	3.2643 (19)	168
C3—H3*A*⋯*Cg*2^ii^	0.97	2.85	3.797 (2)	166

Symmetry codes: (i) 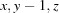; (ii) 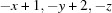.

**Table 3:** Experimental details

	**1**	**2**
Crystal data		
Chemical formula	C_10_H_8_Cl_3_NOS	C_10_H_7_Cl_4_NOS
*M*_r_	296.58	331.03
Crystal system, space group	Triclinic, *P* 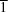	Monoclinic, *P*2_1_/*n*
Temperature (K)	298	298
*a*, *b*, *c* (Å)	6.1968 (13), 9.578 (2), 10.854 (2)	10.907 (2), 6.1686 (12), 19.885 (4)
α, β, γ (°)	103.135 (4), 91.319 (3), 99.239 (3)	90, 96.02 (3), 90
*V* (Å^3^)	618.0 (2)	1330.5 (5)
*Z*	2	4
Radiation type	Mo *K*α	Mo *K*α
μ (mm^−1^)	0.89	1.03
Crystal size (mm)	0.3 × 0.05 × 0.03	0.25 × 0.13 × 0.1

Data collection		
Diffractometer	Bruker SMART CCD area detector	Bruker SMART CCD area detector
Absorption correction	Multi-scan (*SADABS*; Bruker, 2001 ▸)	Multi-scan (*SADABS*; BRUKER, 2001 ▸)
*T*_min_, *T*_max_	0.769, 0.9	0.868, 0.9
No. of measured, independent and observed [*I* > 2σ(*I*)] reflections	5921, 2977, 2634	12273, 3302, 2883
*R*_int_	0.016	0.018
(sin θ/λ)_max_ (Å^−1^)	0.667	0.668

Refinement		
*R*[*F* ^2^ > 2σ(*F* ^2^)], *wR*(*F* ^2^), *S*	0.038, 0.118, 1.01	0.036, 0.107, 1.00
No. of reflections	2977	3302
No. of parameters	145	154
H-atom treatment	H-atom parameters constrained	H-atom parameters constrained
Δρ_max_, Δρ_min_ (e Å^−3^)	0.53, −0.47	0.47, −0.25

Computer programs: *SMART* and *SAINT* (Bruker, 2001 ▸), *olex2.solve* (Bourhis *et al.*, 2015 ▸), *SHELXS* and *SHELXL* (Sheldrick, 2008 ▸) and *OLEX2* (Dolomanov *et al.*, 2009 ▸).

## supplementary crystallographic information

### 2-Trichloromethyl-3-phenyl-1,3-thiazolidin-4-one (1) Crystal data

**Table:**

C_10_H_8_Cl_3_NOS	*Z* = 2
*M**_r_* = 296.58	*F*(000) = 300
Triclinic, *P*1	*D*_x_ = 1.594 Mg m^−^^3^
*a* = 6.1968 (13) Å	Mo *K*α radiation, λ = 0.71073 Å
*b* = 9.578 (2) Å	Cell parameters from 3362 reflections
*c* = 10.854 (2) Å	θ = 2.2–28.3°
α = 103.135 (4)°	µ = 0.89 mm^−^^1^
β = 91.319 (3)°	*T* = 298 K
γ = 99.239 (3)°	Needle, colorless
*V* = 618.0 (2) Å^3^	0.3 × 0.05 × 0.03 mm

### 2-Trichloromethyl-3-phenyl-1,3-thiazolidin-4-one (1) Data collection

**Table:**

Bruker SMART CCD area detector diffractometer	2977 independent reflections
Radiation source: fine-focus sealed tube	2634 reflections with *I* > 2σ(*I*)
Parallel,graphite monochromator	*R*_int_ = 0.016
phi and ω scans	θ_max_ = 28.3°, θ_min_ = 1.9°
Absorption correction: multi-scan (SADABS; Bruker, 2001)	*h* = −8→8
*T*_min_ = 0.769, *T*_max_ = 0.9	*k* = −12→12
5921 measured reflections	*l* = −14→13

### 2-Trichloromethyl-3-phenyl-1,3-thiazolidin-4-one (1) Refinement

**Table:**

Refinement on *F*^2^	Primary atom site location: iterative
Least-squares matrix: full	Secondary atom site location: difference Fourier map
*R*[*F*^2^ > 2σ(*F*^2^)] = 0.038	Hydrogen site location: inferred from neighbouring sites
*wR*(*F*^2^) = 0.118	H-atom parameters constrained
*S* = 1.01	*w* = 1/[σ^2^(*F*_o_^2^) + (0.0658*P*)^2^ + 0.2774*P*] where *P* = (*F*_o_^2^ + 2*F*_c_^2^)/3
2977 reflections	(Δ/σ)_max_ < 0.001
145 parameters	Δρ_max_ = 0.53 e Å^−^^3^
0 restraints	Δρ_min_ = −0.47 e Å^−^^3^

### 2-Trichloromethyl-3-phenyl-1,3-thiazolidin-4-one (1) Special details

**Table:**

Experimental. The data collection nominally covered a full sphere of reciprocal space by a combination of 4 sets of ω scans each set at different φ and/or 2θ angles and each scan (20 s exposure) covering -0.300° degrees in ω. The crystal to detector distance was 5.82 cm.(SADABS; Bruker, 2001) was used for absorption correction. R(int) was 0.0816 before and 0.0197 after correction. The Ratio of minimum to maximum transmission is 0.7686. The λ/2 correction factor is 0.0015.
Geometry. All esds (except the esd in the dihedral angle between two l.s. planes) are estimated using the full covariance matrix. The cell esds are taken into account individually in the estimation of esds in distances, angles and torsion angles; correlations between esds in cell parameters are only used when they are defined by crystal symmetry. An approximate (isotropic) treatment of cell esds is used for estimating esds involving l.s. planes.
Refinement. Refinement of F^2^ against ALL reflections. The weighted R-factor wR and goodness of fit S are based on F^2^, conventional R-factors R are based on F, with F set to zero for negative F^2^. The threshold expression of F^2^ > 2sigma(F^2^) is used only for calculating R-factors(gt) etc. and is not relevant to the choice of reflections for refinement. R-factors based on F^2^ are statistically about twice as large as those based on F, and R- factors based on ALL data will be even larger.

### 2-Trichloromethyl-3-phenyl-1,3-thiazolidin-4-one (1) Fractional atomic coordinates and isotropic or equivalent isotropic displacement parameters (Å^2^)

**Table:**

	*x*	*y*	*z*	*U*_iso_*/*U*_eq_	
Cl1	0.06476 (9)	0.12635 (6)	0.75819 (5)	0.05390 (17)	
Cl3	0.50432 (10)	0.09734 (7)	0.68826 (7)	0.0654 (2)	
Cl2	0.42957 (13)	0.34274 (7)	0.87998 (6)	0.0692 (2)	
S1	0.22368 (9)	0.18205 (6)	0.47724 (5)	0.04902 (16)	
O1	−0.1907 (2)	0.43408 (17)	0.58501 (16)	0.0491 (4)	
N1	0.1467 (2)	0.40320 (17)	0.65276 (15)	0.0367 (3)	
C3	−0.0461 (3)	0.2263 (2)	0.4763 (2)	0.0469 (5)	
H3A	−0.0878	0.2397	0.3937	0.056*	
H3B	−0.1511	0.1483	0.4950	0.056*	
C2	−0.0430 (3)	0.3648 (2)	0.57597 (18)	0.0377 (4)	
C1	0.3059 (3)	0.3068 (2)	0.62984 (18)	0.0369 (4)	
H1	0.4498	0.3643	0.6244	0.044*	
C5	0.1956 (3)	0.5430 (2)	0.73953 (18)	0.0399 (4)	
C10	0.0444 (4)	0.5876 (3)	0.8246 (2)	0.0599 (6)	
H10	−0.0856	0.5257	0.8292	0.072*	
C9	0.0893 (6)	0.7262 (4)	0.9031 (3)	0.0798 (9)	
H9	−0.0133	0.7584	0.9592	0.096*	
C8	0.2836 (6)	0.8162 (3)	0.8989 (3)	0.0743 (8)	
H8	0.3127	0.9086	0.9526	0.089*	
C7	0.4339 (5)	0.7705 (3)	0.8162 (3)	0.0609 (6)	
H7	0.5660	0.8316	0.8145	0.073*	
C6	0.3914 (4)	0.6338 (2)	0.7347 (2)	0.0467 (4)	
H6	0.4932	0.6033	0.6774	0.056*	
C4	0.3226 (3)	0.2231 (2)	0.7350 (2)	0.0420 (4)	

### 2-Trichloromethyl-3-phenyl-1,3-thiazolidin-4-one (1) Atomic displacement parameters (Å^2^)

**Table:**

	*U*^11^	*U*^22^	*U*^33^	*U*^12^	*U*^13^	*U*^23^
Cl1	0.0461 (3)	0.0603 (3)	0.0560 (3)	−0.0005 (2)	0.0048 (2)	0.0215 (2)
Cl3	0.0508 (3)	0.0625 (4)	0.0953 (5)	0.0280 (3)	0.0029 (3)	0.0312 (3)
Cl2	0.0806 (4)	0.0620 (4)	0.0589 (4)	0.0015 (3)	−0.0314 (3)	0.0126 (3)
S1	0.0514 (3)	0.0515 (3)	0.0447 (3)	0.0196 (2)	0.0057 (2)	0.0045 (2)
O1	0.0336 (7)	0.0512 (8)	0.0644 (9)	0.0133 (6)	−0.0008 (6)	0.0138 (7)
N1	0.0307 (7)	0.0353 (8)	0.0443 (8)	0.0086 (6)	0.0001 (6)	0.0076 (6)
C3	0.0442 (10)	0.0450 (11)	0.0498 (11)	0.0091 (8)	−0.0080 (8)	0.0072 (9)
C2	0.0317 (8)	0.0383 (9)	0.0448 (10)	0.0058 (7)	0.0017 (7)	0.0133 (7)
C1	0.0302 (8)	0.0369 (9)	0.0450 (9)	0.0079 (7)	0.0021 (7)	0.0108 (7)
C5	0.0437 (9)	0.0370 (9)	0.0399 (9)	0.0102 (8)	−0.0011 (7)	0.0091 (7)
C10	0.0561 (13)	0.0633 (14)	0.0547 (13)	0.0111 (11)	0.0109 (10)	0.0010 (11)
C9	0.090 (2)	0.080 (2)	0.0598 (16)	0.0294 (17)	0.0101 (15)	−0.0135 (14)
C8	0.098 (2)	0.0517 (14)	0.0621 (16)	0.0133 (15)	−0.0183 (15)	−0.0089 (12)
C7	0.0715 (15)	0.0427 (12)	0.0623 (14)	−0.0014 (11)	−0.0179 (12)	0.0095 (10)
C6	0.0486 (11)	0.0407 (10)	0.0512 (11)	0.0051 (8)	−0.0028 (9)	0.0136 (9)
C4	0.0339 (8)	0.0432 (10)	0.0502 (11)	0.0063 (7)	−0.0044 (7)	0.0143 (8)

### 2-Trichloromethyl-3-phenyl-1,3-thiazolidin-4-one (1) Geometric parameters (Å, º)

**Table:**

Cl1—C4	1.767 (2)	C1—C4	1.547 (3)
Cl3—C4	1.778 (2)	C5—C10	1.379 (3)
Cl2—C4	1.766 (2)	C5—C6	1.384 (3)
S1—C3	1.790 (2)	C10—H10	0.9300
S1—C1	1.816 (2)	C10—C9	1.386 (4)
O1—C2	1.208 (2)	C9—H9	0.9300
N1—C2	1.374 (2)	C9—C8	1.372 (5)
N1—C1	1.445 (2)	C8—H8	0.9300
N1—C5	1.434 (2)	C8—C7	1.362 (4)
C3—H3A	0.9700	C7—H7	0.9300
C3—H3B	0.9700	C7—C6	1.386 (3)
C3—C2	1.506 (3)	C6—H6	0.9300
C1—H1	0.9800		
			
C3—S1—C1	92.98 (9)	C5—C10—H10	120.5
C2—N1—C1	117.34 (16)	C5—C10—C9	119.0 (3)
C2—N1—C5	120.50 (15)	C9—C10—H10	120.5
C5—N1—C1	121.60 (15)	C10—C9—H9	119.7
S1—C3—H3A	110.2	C8—C9—C10	120.6 (3)
S1—C3—H3B	110.2	C8—C9—H9	119.7
H3A—C3—H3B	108.5	C9—C8—H8	119.9
C2—C3—S1	107.58 (14)	C7—C8—C9	120.1 (2)
C2—C3—H3A	110.2	C7—C8—H8	119.9
C2—C3—H3B	110.2	C8—C7—H7	119.8
O1—C2—N1	124.51 (18)	C8—C7—C6	120.5 (3)
O1—C2—C3	123.05 (18)	C6—C7—H7	119.8
N1—C2—C3	112.43 (16)	C5—C6—C7	119.2 (2)
S1—C1—H1	108.8	C5—C6—H6	120.4
N1—C1—S1	106.52 (12)	C7—C6—H6	120.4
N1—C1—H1	108.8	Cl1—C4—Cl3	108.87 (11)
N1—C1—C4	112.89 (16)	Cl2—C4—Cl1	108.94 (12)
C4—C1—S1	110.96 (14)	Cl2—C4—Cl3	108.22 (10)
C4—C1—H1	108.8	C1—C4—Cl1	111.51 (13)
C10—C5—N1	119.72 (19)	C1—C4—Cl3	108.20 (14)
C10—C5—C6	120.6 (2)	C1—C4—Cl2	111.02 (14)
C6—C5—N1	119.70 (18)		
			
S1—C3—C2—O1	168.28 (16)	C1—S1—C3—C2	15.36 (16)
S1—C3—C2—N1	−10.8 (2)	C1—N1—C2—O1	179.29 (18)
S1—C1—C4—Cl1	−64.40 (16)	C1—N1—C2—C3	−1.7 (2)
S1—C1—C4—Cl3	55.31 (15)	C1—N1—C5—C10	−135.2 (2)
S1—C1—C4—Cl2	173.93 (9)	C1—N1—C5—C6	46.7 (3)
N1—C1—C4—Cl1	55.1 (2)	C5—N1—C2—O1	−9.2 (3)
N1—C1—C4—Cl3	174.80 (12)	C5—N1—C2—C3	169.87 (17)
N1—C1—C4—Cl2	−66.58 (18)	C5—N1—C1—S1	−158.41 (14)
N1—C5—C10—C9	−176.5 (2)	C5—N1—C1—C4	79.6 (2)
N1—C5—C6—C7	177.9 (2)	C5—C10—C9—C8	−1.8 (5)
C3—S1—C1—N1	−16.08 (14)	C10—C5—C6—C7	−0.2 (3)
C3—S1—C1—C4	107.16 (14)	C10—C9—C8—C7	0.7 (5)
C2—N1—C1—S1	13.0 (2)	C9—C8—C7—C6	0.7 (4)
C2—N1—C1—C4	−108.99 (19)	C8—C7—C6—C5	−1.0 (4)
C2—N1—C5—C10	53.6 (3)	C6—C5—C10—C9	1.5 (4)
C2—N1—C5—C6	−124.5 (2)		

### 2-Trichloromethyl-3-phenyl-1,3-thiazolidin-4-one (1) Hydrogen-bond geometry (Å, º)

Cg2 is the centroid of the C5–C10 ring

**Table:**

*D*—H···*A*	*D*—H	H···*A*	*D*···*A*	*D*—H···*A*
C1—H1···O1^i^	0.98	2.30	3.251 (2)	164
C3—H3*A*···*Cg*2^ii^	0.97	2.79	3.748 (2)	170

Symmetry codes: (i) *x*+1, *y*, *z*; (ii) −*x*, −*y*+1, −*z*+1.

### 3-(4-Chlorophenyl)-2-trichloromethyl-1,3-thiazolidin-4-one (2) Crystal data

**Table:**

C_10_H_7_Cl_4_NOS	*F*(000) = 664
*M**_r_* = 331.03	*D*_x_ = 1.653 Mg m^−^^3^
Monoclinic, *P*2_1_/*n*	Mo *K*α radiation, λ = 0.71073 Å
*a* = 10.907 (2) Å	Cell parameters from 5450 reflections
*b* = 6.1686 (12) Å	θ = 2.2–28.3°
*c* = 19.885 (4) Å	µ = 1.03 mm^−^^1^
β = 96.02 (3)°	*T* = 298 K
*V* = 1330.5 (5) Å^3^	Block, colorless
*Z* = 4	0.25 × 0.13 × 0.1 mm

### 3-(4-Chlorophenyl)-2-trichloromethyl-1,3-thiazolidin-4-one (2) Data collection

**Table:**

Bruker SMART CCD area detector diffractometer	3302 independent reflections
Radiation source: fine-focus sealed tube	2883 reflections with *I* > 2σ(*I*)
Graphite monochromator	*R*_int_ = 0.018
ω scans	θ_max_ = 28.3°, θ_min_ = 2.0°
Absorption correction: multi-scan (SADABS; BRUKER, 2001)	*h* = −12→14
*T*_min_ = 0.868, *T*_max_ = 0.9	*k* = −8→8
12273 measured reflections	*l* = −26→23

### 3-(4-Chlorophenyl)-2-trichloromethyl-1,3-thiazolidin-4-one (2) Refinement

**Table:**

Refinement on *F*^2^	Primary atom site location: structure-invariant direct methods
Least-squares matrix: full	Secondary atom site location: difference Fourier map
*R*[*F*^2^ > 2σ(*F*^2^)] = 0.036	Hydrogen site location: inferred from neighbouring sites
*wR*(*F*^2^) = 0.107	H-atom parameters constrained
*S* = 1.00	*w* = 1/[σ^2^(*F*_o_^2^) + (0.069*P*)^2^ + 0.2954*P*] where *P* = (*F*_o_^2^ + 2*F*_c_^2^)/3
3302 reflections	(Δ/σ)_max_ < 0.001
154 parameters	Δρ_max_ = 0.47 e Å^−^^3^
0 restraints	Δρ_min_ = −0.25 e Å^−^^3^

### 3-(4-Chlorophenyl)-2-trichloromethyl-1,3-thiazolidin-4-one (2) Special details

**Table:**

Experimental. The data collection nominally covered a full sphere of reciprocal space by a combination of 4 sets of ω scans each set at different φ and/or 2θ angles and each scan (10 s exposure) covering -0.300° degrees in ω. The crystal to detector distance was 5.82 cm.SADABS V2.05 (BRUKER, 2001) was used for absorption correction. R(int) was 0.0443 before and 0.0167 after correction. The Ratio of minimum to maximum transmission is 0.8678. The λ/2 correction factor is 0.0015.
Geometry. All esds (except the esd in the dihedral angle between two l.s. planes) are estimated using the full covariance matrix. The cell esds are taken into account individually in the estimation of esds in distances, angles and torsion angles; correlations between esds in cell parameters are only used when they are defined by crystal symmetry. An approximate (isotropic) treatment of cell esds is used for estimating esds involving l.s. planes.
Refinement. Refinement of F^2^ against ALL reflections. The weighted R-factor wR and goodness of fit S are based on F^2^, conventional R-factors R are based on F, with F set to zero for negative F^2^. The threshold expression of F^2^ > 2sigma(F^2^) is used only for calculating R-factors(gt) etc. and is not relevant to the choice of reflections for refinement. R-factors based on F^2^ are statistically about twice as large as those based on F, and R- factors based on ALL data will be even larger.

### 3-(4-Chlorophenyl)-2-trichloromethyl-1,3-thiazolidin-4-one (2) Fractional atomic coordinates and isotropic or equivalent isotropic displacement parameters (Å^2^)

**Table:**

	*x*	*y*	*z*	*U*_iso_*/*U*_eq_	
S1	0.57569 (4)	0.69974 (7)	0.15220 (2)	0.04596 (13)	
Cl1	0.87252 (5)	0.86052 (9)	0.16992 (3)	0.05996 (16)	
Cl3	0.81425 (5)	0.41241 (9)	0.19023 (3)	0.06369 (16)	
Cl2	0.92398 (5)	0.53959 (10)	0.07182 (3)	0.06652 (18)	
Cl4	0.85656 (7)	0.75038 (16)	−0.22386 (3)	0.0903 (2)	
N1	0.67812 (13)	0.8295 (2)	0.04570 (6)	0.0375 (3)	
C5	0.72089 (15)	0.8111 (3)	−0.01992 (8)	0.0379 (3)	
C4	0.81846 (16)	0.6202 (3)	0.12933 (8)	0.0407 (3)	
C1	0.68725 (15)	0.6485 (2)	0.09226 (7)	0.0356 (3)	
H1	0.6640	0.5154	0.0672	0.043*	
C2	0.61543 (16)	1.0100 (3)	0.06349 (8)	0.0412 (3)	
O1	0.60428 (13)	1.17453 (19)	0.03021 (7)	0.0531 (3)	
C8	0.80220 (18)	0.7763 (4)	−0.14533 (9)	0.0555 (5)	
C6	0.68763 (17)	0.6339 (3)	−0.05979 (8)	0.0446 (4)	
H6	0.6373	0.5273	−0.0443	0.053*	
C3	0.56058 (19)	0.9806 (3)	0.12923 (10)	0.0507 (4)	
H3A	0.4743	1.0222	0.1240	0.061*	
H3B	0.6037	1.0704	0.1641	0.061*	
C10	0.79363 (19)	0.9722 (3)	−0.04301 (10)	0.0555 (5)	
H10	0.8158	1.0918	−0.0160	0.067*	
C9	0.8334 (2)	0.9550 (4)	−0.10653 (11)	0.0657 (6)	
H9	0.8811	1.0642	−0.1228	0.079*	
C7	0.72899 (18)	0.6145 (3)	−0.12273 (9)	0.0518 (4)	
H7	0.7079	0.4941	−0.1496	0.062*	

### 3-(4-Chlorophenyl)-2-trichloromethyl-1,3-thiazolidin-4-one (2) Atomic displacement parameters (Å^2^)

**Table:**

	*U*^11^	*U*^22^	*U*^33^	*U*^12^	*U*^13^	*U*^23^
S1	0.0473 (3)	0.0496 (2)	0.0431 (2)	−0.00669 (17)	0.01474 (18)	−0.00091 (17)
Cl1	0.0493 (3)	0.0606 (3)	0.0695 (3)	−0.0120 (2)	0.0035 (2)	−0.0186 (2)
Cl3	0.0798 (4)	0.0595 (3)	0.0508 (3)	0.0090 (2)	0.0024 (2)	0.0189 (2)
Cl2	0.0631 (3)	0.0876 (4)	0.0511 (3)	0.0291 (3)	0.0168 (2)	−0.0008 (2)
Cl4	0.0881 (5)	0.1389 (6)	0.0497 (3)	0.0127 (4)	0.0347 (3)	0.0164 (3)
N1	0.0477 (7)	0.0317 (6)	0.0336 (6)	−0.0006 (5)	0.0070 (5)	0.0000 (5)
C5	0.0417 (8)	0.0386 (7)	0.0338 (7)	−0.0018 (6)	0.0058 (6)	0.0037 (6)
C4	0.0480 (9)	0.0404 (8)	0.0346 (8)	0.0026 (6)	0.0082 (6)	0.0001 (6)
C1	0.0449 (8)	0.0315 (6)	0.0307 (7)	−0.0035 (6)	0.0063 (6)	−0.0016 (5)
C2	0.0457 (8)	0.0349 (7)	0.0419 (8)	−0.0017 (6)	−0.0005 (6)	−0.0047 (6)
O1	0.0675 (9)	0.0331 (6)	0.0581 (8)	0.0029 (5)	0.0030 (6)	0.0019 (5)
C8	0.0502 (10)	0.0796 (13)	0.0390 (9)	0.0082 (9)	0.0155 (8)	0.0131 (9)
C6	0.0533 (10)	0.0455 (8)	0.0360 (8)	−0.0065 (7)	0.0098 (7)	0.0006 (6)
C3	0.0551 (10)	0.0501 (9)	0.0477 (10)	0.0101 (8)	0.0087 (8)	−0.0055 (8)
C10	0.0612 (11)	0.0503 (10)	0.0567 (11)	−0.0161 (8)	0.0139 (9)	0.0015 (8)
C9	0.0635 (13)	0.0725 (13)	0.0650 (13)	−0.0143 (10)	0.0256 (10)	0.0165 (11)
C7	0.0578 (11)	0.0627 (11)	0.0359 (8)	−0.0008 (9)	0.0090 (7)	−0.0040 (8)

### 3-(4-Chlorophenyl)-2-trichloromethyl-1,3-thiazolidin-4-one (2) Geometric parameters (Å, º)

**Table:**

S1—C1	1.8181 (16)	C2—O1	1.211 (2)
S1—C3	1.795 (2)	C2—C3	1.505 (3)
Cl1—C4	1.7595 (17)	C8—C9	1.368 (3)
Cl3—C4	1.7674 (17)	C8—C7	1.382 (3)
Cl2—C4	1.7766 (17)	C6—H6	0.9300
Cl4—C8	1.7349 (19)	C6—C7	1.379 (2)
N1—C5	1.436 (2)	C3—H3A	0.9700
N1—C1	1.4471 (18)	C3—H3B	0.9700
N1—C2	1.372 (2)	C10—H10	0.9300
C5—C6	1.377 (2)	C10—C9	1.382 (3)
C5—C10	1.380 (2)	C9—H9	0.9300
C4—C1	1.549 (2)	C7—H7	0.9300
C1—H1	0.9800		
			
C3—S1—C1	92.91 (8)	C9—C8—Cl4	119.59 (16)
C5—N1—C1	120.90 (12)	C9—C8—C7	121.07 (17)
C2—N1—C5	121.10 (13)	C7—C8—Cl4	119.33 (18)
C2—N1—C1	117.64 (13)	C5—C6—H6	120.0
C6—C5—N1	119.66 (14)	C5—C6—C7	119.99 (17)
C6—C5—C10	120.42 (16)	C7—C6—H6	120.0
C10—C5—N1	119.91 (15)	S1—C3—H3A	110.2
Cl1—C4—Cl3	109.17 (9)	S1—C3—H3B	110.2
Cl1—C4—Cl2	108.82 (10)	C2—C3—S1	107.67 (12)
Cl3—C4—Cl2	107.64 (9)	C2—C3—H3A	110.2
C1—C4—Cl1	111.83 (11)	C2—C3—H3B	110.2
C1—C4—Cl3	108.57 (11)	H3A—C3—H3B	108.5
C1—C4—Cl2	110.71 (11)	C5—C10—H10	120.2
S1—C1—H1	108.9	C5—C10—C9	119.67 (19)
N1—C1—S1	106.23 (10)	C9—C10—H10	120.2
N1—C1—C4	112.92 (13)	C8—C9—C10	119.64 (18)
N1—C1—H1	108.9	C8—C9—H9	120.2
C4—C1—S1	110.85 (10)	C10—C9—H9	120.2
C4—C1—H1	108.9	C8—C7—H7	120.4
N1—C2—C3	112.34 (14)	C6—C7—C8	119.18 (19)
O1—C2—N1	124.29 (16)	C6—C7—H7	120.4
O1—C2—C3	123.36 (16)		
			
Cl1—C4—C1—S1	−65.17 (12)	C1—S1—C3—C2	15.17 (13)
Cl1—C4—C1—N1	53.89 (15)	C1—N1—C5—C6	47.7 (2)
Cl3—C4—C1—S1	55.34 (12)	C1—N1—C5—C10	−133.18 (17)
Cl3—C4—C1—N1	174.40 (10)	C1—N1—C2—O1	177.64 (15)
Cl2—C4—C1—S1	173.30 (8)	C1—N1—C2—C3	−2.8 (2)
Cl2—C4—C1—N1	−67.64 (14)	C2—N1—C5—C6	−125.20 (17)
Cl4—C8—C9—C10	−177.79 (18)	C2—N1—C5—C10	54.0 (2)
Cl4—C8—C7—C6	178.93 (15)	C2—N1—C1—S1	13.99 (17)
N1—C5—C6—C7	−179.61 (16)	C2—N1—C1—C4	−107.72 (16)
N1—C5—C10—C9	−179.26 (18)	O1—C2—C3—S1	169.53 (14)
N1—C2—C3—S1	−10.00 (18)	C6—C5—C10—C9	−0.1 (3)
C5—N1—C1—S1	−159.12 (12)	C3—S1—C1—N1	−16.38 (12)
C5—N1—C1—C4	79.18 (17)	C3—S1—C1—C4	106.63 (12)
C5—N1—C2—O1	−9.3 (3)	C10—C5—C6—C7	1.2 (3)
C5—N1—C2—C3	170.25 (14)	C9—C8—C7—C6	−0.4 (3)
C5—C6—C7—C8	−1.0 (3)	C7—C8—C9—C10	1.5 (3)
C5—C10—C9—C8	−1.3 (3)		

### 3-(4-Chlorophenyl)-2-trichloromethyl-1,3-thiazolidin-4-one (2) Hydrogen-bond geometry (Å, º)

Cg2 is the centroid of the C5–C10 ring

**Table:**

*D*—H···*A*	*D*—H	H···*A*	*D*···*A*	*D*—H···*A*
C1—H1···O1^i^	0.98	2.30	3.2643 (19)	168
C3—H3*A*···*Cg*2^ii^	0.97	2.85	3.797 (2)	166

Symmetry codes: (i) *x*, *y*−1, *z*; (ii) −*x*+1, −*y*+2, −*z*.
